# Improved Asymptotic Friis Formula with Higher-Order Terms: Calculating Antenna Coupling in Close Proximity

**DOI:** 10.3390/s26154833

**Published:** 2026-07-31

**Authors:** Ilkyu Kim, Jeong-Hae Lee

**Affiliations:** 1 Department of Electrical and Electronics Engineering, Sejong Cyber University, Seoul 05000, Republic of Korea; ikkim0704@sjcu.ac.kr; 2 Department of Electronics and Electrical Engineering, Hongik University, Seoul 04066, Republic of Korea

**Keywords:** near-field communication, antenna coupling, asymptotic gain reduction factor, higher-order terms, circular aperture, rectangular aperture, power transmission

## Abstract

The emergence of near-field communication as a part of sixth-generation (6G) communication necessitates accurate estimation of the link budget between two antennas operating in the near-field region. In this paper, we present an asymptotic expression for the gain reduction factor that includes higher-order terms to evaluate the accuracy of predicting antenna coupling at short distances in the near-field region. Asymptotic Friis formulas with gain reduction factors for both circular and rectangular apertures are examined, and we show that asymptotic formulas including higher-order terms yield a 22–25% shorter minimum prediction distance than those with lower-order terms. The validity of the formula is evaluated through the full-wave simulation and measurement.

## 1. Introduction

There have been recent advancements towards large arrays for a massive MIMO system, which can be essential for sixth-generation communications [1,2,3]. As the electrical sizes of antennas increase, these antennas are increasingly being used at near-field distances. Determining the coupling between two antennas in the near-field region has been more emphasized in recent years than in the past [4,5,6], when the far-field communication was prevalent. Some emerging applications that operate in the near-field region include wireless power transfer (WPT), wireless medical telemetry, and imaging [7,8,9]. For these applications, it is critical to reduce interference with other users and increase the operating range of the devices. In order to achieve the design goal, it is required to use an appropriate method to predict the power transmission level between two closely spaced antennas.

Several efforts have been made in order to accurately estimate the power transmission within a radiating near-field region. The integral form of the coupling formula has been widely used to calculate the wireless link budget for a variety of applications, including small antennas for pacemakers [9]. Based on the integral of the scalar product of the two far-field radiation patterns, it can offer mutual coupling for numerous possible antenna configurations [10,11,12,13]. However, the integral coupling requires a relatively complex computation based on the three-dimensional (3D) far-field patterns of the two antennas. Another option is full-wave simulation, which has been widely used to evaluate the mutual coupling between two antennas due to its high precision, independent of antenna configuration. However, it involves much more intense computation, which should be repeated for each antenna movement. The Friis power transmission formula is a well-known and widely used expression due to its simplicity and usefulness [14]. The formula has been derived using four different approaches—effective aperture area, Fresnel optics, Gaussian beam, and mode analysis—all of which are based on the plane waves. These four methods assume that the formula is valid only in the far-field region. Since the antenna gain in the near-field region differs from that in the far-field region, the accurate variation in the near-field gain should be considered. Several attempts have been made to accurately estimate the antenna gain that may vary in the Fresnel region [15,16,17,18]. The near-field gain has been represented as the product of the far-field gain and a correction term operating within the near-field region. The notion of a gain reduction factor has been introduced to predict the Fresnel gain of antennas. There are different methods for deriving the gain reduction factor using the Sommerfeld series solution [15] and the Fresnel integral [16]. However, all of them utilize the normalization based on the aperture diameter *D* and separation distance *R*. There have been advances in presenting a gain reduction factor for a wider range of antennas by normalizing the separation distance with far-field gain and wavelength [18]. One limitation of such work is that accuracy decreases at very close distances in the Fresnel region due to its quadratic form. The formula has been generalized to include the multi-port antenna system [5]. The formulas based on the power balance theory (PBT) and beam efficiency (BE) provide explicit expressions for estimating the coupling in both the far- and near-field region [19,20].

In this paper, the asymptotic gain reduction factor using higher-order terms is studied in order to extend the effective prediction range of antenna coupling. The asymptotic gain reduction factor is advantageous because of its simplicity in use and effectiveness in terms of its operating range. In addition, the gain reduction factor is useful because it offers physical insight into the behavior of Tx and Rx antenna in both near- and far-field regions. The gain reduction factors for circular and rectangular apertures are obtained through the Taylor expansion of the analytical solution presented in [15]. Compared with our preliminary work [21,22], the present paper provides several extensions. The different gain reduction factor behaviors of circular and rectangular apertures are discussed. The effect of including a higher-order term is studied in terms of the accuracy and range of the formula. The power transmission obtained using different orders of the asymptotic formula is compared with the full-wave simulation and measurement.

## 2. Materials and Methods

One antenna is placed in the near-field or far-field region of the other antenna. The power transmission between the two antennas can be defined as the ratio between a transmitted power *P*_t_ and a received power *P*_r_ using the Friis formula presented in [14,18]:

(1) $${\left|S_{21}(R,\;\theta_{t}{,\;\varphi }_{t})\right|}^{2}=\frac{P_{r}}{P_{t}}=\frac{\lambda^{2}}{16\pi^{2}R^{2}}G_{t}(\theta_{t}{,\;\varphi }_{t})G_{r}(\theta_{t}{,\;\varphi }_{t})$$

where $G_{t}(\theta_{t}{,\;\varphi }_{t})$ and $G_{r}(\theta_{t}{,\;\varphi }_{t})$ are far-field patterns of transmitting and receiving antenna, respectively. This work is mainly focused on the boresight power transmission under polarization-matched conditions. Far-field broadside gain is used instead of the far-field pattern. *R* represents the distance between the phase centers of the two antennas. For lateral and angular offset displacement, another correction term to the far-field pattern can be used to approximate the angular variation as *R* varies within the Fresnel region. The power transmission between two antennas in the near-field region shows considerable deviation compared to that obtained using the standard Friis formula. This is attributed to the fact that the antenna gain in the near-field region is as a function of distance *R*, while that of the far-field is constant regardless of the distance *R*. In order to enhance the accuracy in the near-field region, a correction term, called a gain reduction factor, can be incorporated into the Friis formula:

(2) $$\frac{P_{r}}{P_{t}}=\frac{\lambda^{2}}{16\pi^{2}R^{2}}G_{t}{\;\gamma }_{t}G_{r}{\;\gamma }_{r}$$

where ${\;\gamma }_{t}$ and $\gamma_{r}$ are the gain reduction factors of the transmitting and receiving antennas, respectively. The gain reduction factor presented in [15,16,18] is used to derive the asymptotic gain reduction factor.

The gain reduction factor for the circular aperture $\gamma_{cir}$ [16] can be written as

(3) $$\gamma_{cir}(∆)={\left(\frac{sin\;∆}{∆}\right)}^{2}$$

where ∆ is the normalized distance with ${2D}^{2}/\lambda$. For rectangular aperture, the gain reduction factor can be obtained through the Fresnel integral $C\left(v\right)$ and $S\left(v\right)$ as

(4) $$C\left(v\right)=\int_{0}^{v}cos\frac{\pi }{2}t^{2}dt$$

(5) $$S\left(v\right)=\int_{0}^{v}sin\frac{\pi }{2}t^{2}dt$$

where $v=\sqrt{D^{2}/(2R\lambda )}$. Based on the Fresnel integral, the gain reduction factor $\gamma_{rec}$ [16] can be expressed as

(6) $$\gamma_{rec}(v)={\left(\frac{C^{2}(v)+S^{2}(v)}{v^{2}}\right)}^{2}$$

The quadratic approximation has been used to determine the antenna gain in the near-field region [16]; however, in order to extend the effective prediction range of antenna coupling, we study the asymptotic gain reduction factor using higher-order terms.

**Figure 1:**
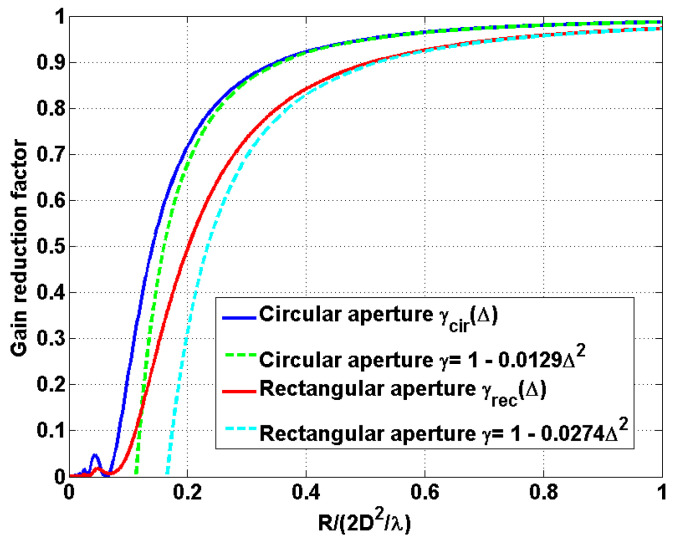
Comparison of gain reduction factors for circular and rectangular apertures.

The asymptotic gain reduction factor is presented employing the Taylor expansion method:

(7) $$\gamma_{2}\left(\Delta \right)=1-\alpha \Delta^{-2}+O\;(\Delta^{-4})$$

(8) $$\gamma_{4}\left(\Delta \right)=1-\alpha \Delta^{-2}+\beta \Delta^{-4}-O(\Delta^{-6})$$

(9) $$\gamma_{6}\left(\Delta \right)=1-\alpha \Delta^{-2}+\beta \Delta^{-4}-\delta \Delta^{-6}\;+O(\Delta^{-10})\;\;\;\;\;\;\;$$

where α, β, and δ refer to coefficients that control variation in the gain reduction factor, and $\Delta =R/{(2D}^{2}/\lambda )$. The lower indexes 2, 4, and 6 of γ(Δ) indicate different orders of the asymptotic expression. The coefficients are given as α = 1.29 × 10^−2^, β = 6.61 × 10^−5^, and δ = 1.82 × 10^−7^ for the circular aperture, and α = 2.74 × 10^−2^, β = 3.48 × 10^−4^, and δ = 2.69 × 10^−6^ for the rectangular aperture. The asymptotic expressions for circular and rectangular aperture are acquired by replacing Equations (3) and (6) with their Taylor expansion. Figure 1 compares the gain reduction factors of the circular and rectangular apertures, assuming that both apertures are based on the same diameter *D*. It is shown that the curve of the rectangular aperture lies below that of the circular aperture, which is consistent with the result reported in [18].

## 3. Results

The different orders of the gain reduction factor are evaluated in terms of the effective range of the asymptotic expression. The baseline power transmission between two antennas is obtained and evaluated with the full-wave simulation, FEKO (Altair Engineering, Troy, MI, USA) and measurement.

### 3.1. Different Orders of Asymptotic Gain Reduction Factor

**Figure 2:**
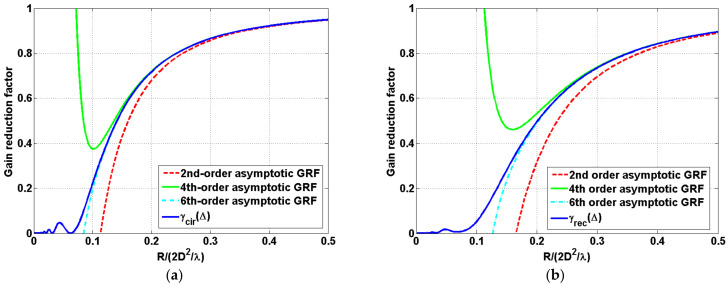
Effect of different orders of asymptotic terms on gain reduction factor for (**a**) circular aperture and (**b**) rectangular aperture.

A comparison of different gain reduction factors is shown in Figure 2. The curve of the second order of the gain reduction factor shows a deviation from other curves at relatively far distances of the near-field region; however, the curve of the sixth order of the gain reduction factor agrees well with the gain reduction factor presented in (3) and (6) at closer distances of the near-field region. It is observed that, by increasing the orders of the asymptotic expression, the accuracy is maintained at a shorter distance in the near-field region. As the distance *R* decreases, the inverse of Δ increases, thereby altering the shape of the gain reduction factor. The second term with a minus sign determines the decrease in the curve, and the fourth term with a plus sign indicates a correction to the curve at shorter distances. However, the fourth term becomes excessively large as the distance decreases, resulting in instability in the curve. Therefore, a sixth term with a minus sign is required to provide stability at very short distances *R*.

### 3.2. Asymptotic Friis Formula

**Figure 3:**
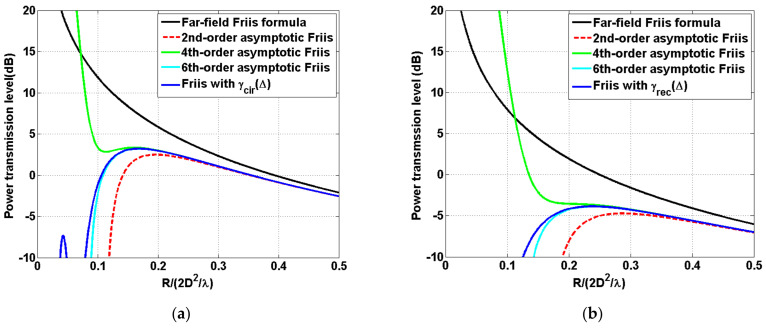
Power transmission using different orders of asymptotic Friis formulas for (**a**) circular aperture and (**b**) rectangular aperture.

The power transmission between two antennas is obtained using the Friis formula incorporated with the gain reduction factor (2). The far-field antenna gain *G*, present in [23], is used to calculate the power transmission:

(10) $$G\;=\frac{4\pi A}{\lambda^{2}}$$

where *A* is the physical area of the aperture antenna. Far-field gains of 33.46 dB and 31.5 dB are obtained using diameter *D* = 15λ for the circular and rectangular apertures, respectively. The power transmissions are computed using the standard Friis formula and a class of the asymptotic Friis formulas. A comparison of the power transmission between two identical circular and rectangular aperture antennas is presented in Figure 3. The first observation is that the standard Friis formula shows good agreement with a group of asymptotic formulas in the far-field region; however, with a decreasing separation distance, it exhibits a large deviation of more than 5 dB from the asymptotic formulas. This represents an improvement obtained through the standard Friis formula. However, the asymptotic formulas decrease rapidly after reaching a certain point in the near-field region, which indicates the failure of the asymptotic expression.

The nearest distances of the effective range are summarized in Table 1. It can be found that closest effective distances that are 22–25% smaller can be achieved using the sixth order of the asymptotic formula, compared to the second order formula. For each model, the minimum applicable distance is defined as the value of *R* corresponding to the peak of the γ curve. Although the fourth-order approximation requires fewer terms, the sixth-order approximation is adopted in this work because it provides more stable and robust predictions. It is demonstrated that the analytical model presented in (3) and (6) agrees well with the sixth-order asymptotic Friis formula.

### 3.3. Full-Wave Simulation and Indoor Measurement

**Figure 4:**
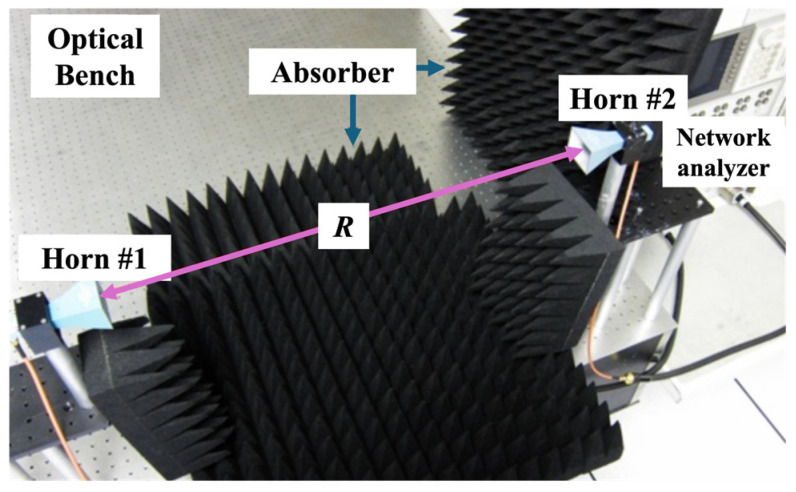
A photograph of the indoor measurement setup adapted from the author’s Ph.D. dissertation.

**Figure 5:**
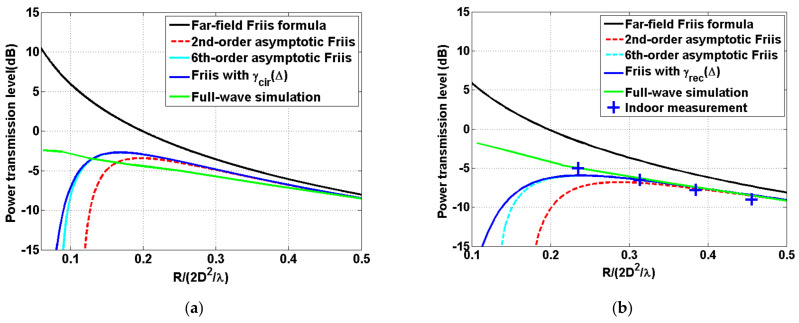
Comparison of full-wave simulation, measurement, and asymptotic Friis formulas for (**a**) circular aperture and (**b**) rectangular aperture.

The result obtained using the proposed formula was evaluated via full-wave simulation FEKO and measurement. The power transmission between two circular apertures was evaluated using two reflector antennas with a radius of 10 λ, which was excited by a feed horn antenna operating at 12.7 GHz. The simulation setup presented in [13] was employed in this work. Power transmission between two reflector antennas was analyzed using FEKO simulations, with the separation distance varying from 50 λ to 1200 λ. For the rectangular aperture antenna case, the measured power transmission between the two identical Ku-band horn antennas presented in [18] was reused in order to validate the proposed formula. The setup of the indoor measurement is shown in Figure 4. Two standard-gain horn antennas with aperture dimensions of 2.15 λ × 1.59 λ were used. An operating frequency of 12.7 GHz was used. The far-field edge distance of ${2D}^{2}/\lambda$ was approximately 14.3 λ. The power transmission levels S_21_ between the two Ku-band horn antennas were obtained by varying the separation distance from 1.5 λ to 15 λ using the full-wave FEKO simulation. The two horn antennas were aligned in the boresight direction, and the power transmission between two antennas was measured using a vector network analyzer (Hewlett-Packard Company, Palo Alto, CA, USA). Both the transmitting and receiving horn antennas were installed at the same height and carefully aligned along the boresight direction. In addition, the optical table was covered with RF absorbers to minimize reflections from the supporting surface. Figure 5 compares the power transmission level between the two Ku-band horn antennas. It is shown that both the full-wave simulation and measured results agree well with the higher-order asymptotic formula. The maximum error of the proposed sixth-order asymptotic formula is within 1.5 dB compared with the full-wave simulation for the circular aperture, and within 1.0 dB compared with the measured results for the rectangular aperture over the applicable range. The full-wave simulation and indoor measurement validate the effectiveness of the formula.

## 4. Discussion

In this article, asymptotic formulas of different orders were studied in terms of their accuracy and effective range. The results indicate that higher-order asymptotic formulas provide better agreement at shorter distances in the near-field region than lower-order formulas. In addition, the full-wave simulation and measured results validate the applicability of the formulas. This work can be extended to various applications, such as reflect-array feed network and near-field communication, where rapid and accurate prediction of wireless power transmission is required.

## Figures and Tables

**Table 1:** Nearest distances of the effective range.

Types of Apertures	2nd Order	6th Order
Circular aperture	0.2 (2*D*^2^/λ)	0.15 (2*D*^2^/λ)
Rectangular aperture	0.28 (2*D*^2^/λ)	0.22 (2*D*^2^/λ)

## Data Availability

The datasets presented in this article are not readily available because the data are part of an ongoing study.

## Abbreviations

The following abbreviations are used in this manuscript:

MIMO	Multiple-Input and Multiple-Output
FEKO	FEldberechnung bei Körpern mit beliebiger Oberfläche
